# Implementation of a clinical pathway for diabetes‐related foot ulcers reduced the number of amputations and shortened hospital stay

**DOI:** 10.1002/jfa2.70024

**Published:** 2025-01-28

**Authors:** Monica Sailer, Hilde Wergeland, Per‐Henrik Randsborg

**Affiliations:** ^1^ Department of Orthopaedic Surgery Akershus University Hospital Lørenskog Norway; ^2^ Institute of Clinical Medicine Faculty of Medicine University of Oslo Oslo Norway; ^3^ Department of Economics and Finance Akershus University Hospital Lørenskog Norway

**Keywords:** diabetic foot ulcer, negative pressure wound therapy, treatment algorithm, vacuum assisted closure, wound care

## Abstract

**Introduction:**

Diabetes‐related foot ulcer (DFU) is the leading cause for lower extremity amputations (LEAs) in western countries, and may cause social isolation, depression, and death. However, people with DFU are not offered the same prioritized care as cancer patients, despite comparable mortality rates. We therefore decided to create a clinical pathway for patients with DFU. The purpose of this study is to evaluate the efficacy of implementing a new clinical pathway on rates of LEA, length of hospital stays, and cost reduction.

**Methods:**

On January 1, 2019, a new clinical pathway ensured that all patients with a DFU were evaluated in a designated clinic run by a foot and ankle orthopedic surgeon in collaboration with the vascular surgeons, supported by a specialized wound nurse and a certified prosthetist/orthotist (CPO). We designed an algorithm for the first consultation to identify patients in need for further investigation by other specialties such as endocrinology, infectious diseases, cardiology, or vascular surgery.

All patients underwent a surgical wound debridement of DFU. Negative pressure wound therapy (NPWT) was not applied. After surgery, the dressings were changed daily on the ward, until the wound was deemed viable and clean. The patients were followed for two years and compared to a historic cohort of patients with DFU admitted to the institution in 2017.

**Results:**

The number of major amputations was reduced from 65% (13/20) to 7.4% (2/27) (*p* < 0.001) after the introduction of the clinical pathway. Both the mean number of surgical revisions (5.5 vs. 1.2) and the median length of stay (46 vs. 9 days) were statistically significantly reduced. The median cost per patient was reduced by 76% (from €538 000 to €129 000, *p* < 0.001).

**Conclusion:**

The clinical pathway for managing DFUs resulted in a reduction in major amputations and shorter hospital stays. Discontinuing NPWT after surgical debridement did not adversely affect clinical outcomes. The new pathway also contributed to lower healthcare costs.

## 1 INTRODUCTION

Diabetes‐related foot ulcer (DFU) is the leading cause of non‐traumatic amputation in the lower extremity in western countries [1]. The 5‐year mortality rate after major lower extremity amputation (LEA) caused by a DFU is 52%–82% [2] which is comparable to cancer [3].

The main cause of the high mortality is an escalation of already established cardiovascular disease, kidney failure, and poor diabetes regulation [4, 5]. In addition, many amputees suffer from social isolation, depression, and lower quality of life [6, 7]. Major LEA is defined as any amputation proximal to the ankle [8].

Several classifications of the diabetic foot provide estimations of the risk of LEA, although none are considered the gold standard [9]. Despite following recommended procedures including adjunct use of negative pressure wound therapy (NPWT), also referred to as vacuum assisted closure [10], we found that patients with DFU had long hospital stays, underwent serial surgical revisions, and still required major LEAs. We suspected that the main reasons for major LEA were under‐treatment of peripheral artery disease and osteomyelitis, probably due to a lack of multidisciplinary approach to the treatment of DFU. Furthermore, we wondered if the use of NPWT in patients with DFU improved outcome to any substantial degree.

Unlike cancer patients, who are enrolled in clinical pathways to secure fast diagnosis and treatment [11], patients suffering from DFU are not offered the same prioritized care, despite comparable mortality rates. We therefore decided to create a clinical pathway for patients with DFU. The purpose of this study is to evaluate the efficacy of implementing a new clinical pathway on rates of LEA, length of hospital stays (LHS), and cost reduction.

## 2 MATERIALS AND METHODS

### 2.1 Clinical pathway

**FIGURE 1:**
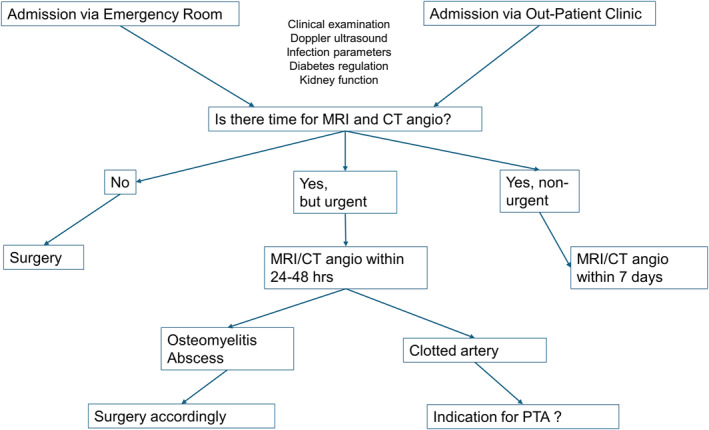
Algorithm for clinical pathway. MRI Magnetic Resonance Imaging, CT Computed tomography, and PTA Percutaneous transluminal angioplasty.

On January 1, 2019, we introduced the following several changes:

A common referral trunk for patients with DFU was created. The new clinical pathway ensured that all patients with a DFU received the same systematic and fast examination (Figure 1). The patients were evaluated in a designated DFU‐clinic run by a foot and ankle orthopedic surgeon in collaboration with the vascular surgeons, supported by a specialized nurse and an orthopedic engineer. We designed an algorithm for the first consultation, to diagnose patients in need for further investigation by other specialties such as endocrinology, cardiology, and vascular surgery. The clinical pathway was based on the guidelines suggested by the International Working Group on the Diabetic Foot [12, 13]. Patients who were admitted acutely via the emergency room were channeled through a similar clinical pathway, with some alterations due to the nature of severe and acute diabetic foot attacks. A diabetic foot attack is characterized by an acutely inflamed foot exhibiting rapidly progressive skin and tissue necrosis, often accompanied by significant systemic symptoms.

Blood samples were collected for the assessment of infection through the measurement of C‐reactive protein (CRP) and white blood cell count (WBC). To monitor glycemic control, glycated hemoglobin (HbA1c) levels were evaluated reflecting average blood glucose levels over the preceding two to 3 months. Additionally, kidney function was assessed using the glomerular filtration rate (GFR) and serum creatinine levels.

All patients underwent a surgical wound debridement of the DFU, with or without additional minor amputations, if required. Minor amputation was defined as any resection through or distal to the ankle, that is, the surgical removal of toes, rays, or segments of the foot [8]. Following comprehensive debridement of the wound or partial amputation of the foot, dressings are applied to maintain a moist wound environment, regulate exudate, prevent maceration of surrounding healthy skin, and promote epithelialization. Routine samples were collected during the surgical procedure, including specimens from the wound for microbiological analysis and from the residual bone for histological examination. Postoperatively, sterile dressings were changed daily in the ward by either a foot and ankle orthopedic surgeon or a designated nurse until the wound was assessed as viable and clean. The objective is to achieve secondary intention healing of the wound. Following the initial healing period, if the wound appears vital and clean, the dressing is transitioned to either a hydrophobic‐coated dressing that binds bacteria and fungi (Sorbact®) or an antibacterial silver dressing (Acticoat Ag7) to cover the wound bed. The surrounding intact skin is protected with sterile cotton compresses or a self‐adherent absorbent dressing (Mepore®). NPWT was not applied.

Following surgery, all patients were administered intravenous cloxacillin or clindamycin in conjunction with ciprofloxacin. The choice of antibiotics was subsequently tailored according to microbiological findings, with a transition from intravenous to oral administration upon clinical and biochemical improvement of infection parameters.

The patients were discharged from the hospital when the wound was deemed suitable for outpatient care. After discharge, the patients attended follow‐up visits at the designated outpatient clinic within a week. The frequency of further follow‐ups were individually adjusted according to the state of the wound and/or the clinical condition of the patient.

### 2.2 Eligibility criteria

Patients with a DFU who were referred to Akershus University Hospital were enrolled in the clinical pathway from January 1 to July 1, 2019, and from January 1 to July 1, 2022. The period of 2020/21 was excluded due to disruptions caused by the pandemic. Patients under the age of 18, with dementia, or severe psychiatric disease were excluded.

The control group comprised a historical cohort of patients diagnosed with a DFU who were admitted to the same institution from January 1 through June 30, 2017. During this period, there was no tailored pathway for patients with DFU. The patients were admitted either via the emergency department or via the outpatient clinic, and were treated by the orthopedic on‐call team. NPWT was frequently used after surgical wound revisions. There were no formalized collaboration with the departments of vascular surgery or endocrinology, and any need for non‐orthopedic treatment was either handled within the orthopedic department or by referral to other departments.

### 2.3 Data collection

Demographic information, clinical data, and resource utilization were gathered from the electronic medical records, prospectively for patients enrolled in the clinical pathway, and retrospectively for the control group.

Outcome variables included length of hospital stay, diagnostic procedures such as computerized tomography angiography (CTA), percutaneous transluminal angioplasty (PTA), and magnetic resonance imaging (MRI), and the number of surgical procedures including major amputations.

### 2.4 Statistical analysis

Descriptive continuous data are presented as mean, 95% confidence interval (CI), and standard deviation (SD), while categorical data is presented in frequencies. Normally distributed variables were compared using student’s *T*‐test and nonparametric variables were compared using the Mann–Whitney *U*‐Test. Categorical data were compared using the chi‐squared test. A *p*‐value of <0.05 was considered significant. The analysis was performed using the statistical package for social sciences (SPSS) version 25 (IBM Corp, Armonk, NY, USA).

### 2.5 Cost per patient (CPP) estimation

The cost estimation of patient treatment was based on elements of the national specification for “cost per patient,” which provides a common standard for calculating costs at the patient level in the healthcare services. The specification forms the basis for the development of CPP models in the institutions [14].

The CPP data reflects the comprehensive costs incurred by the hospital for patient care, which may fluctuate annually. To ensure consistency in our analysis, we utilized unit costs for the year 2022 and adjusted these costs for the years 2017 and 2019 accordingly.

While the CPP model encompasses all service domains within the hospital, our calculations focused specifically on the following selected services:

- Admission route (the orthopedic outpatient clinic or the emergency department)
- Outpatient clinic: Average cost per hour per consultation
- Inpatient ward: Average cost per day at an orthopedic inpatient ward
- Physician service: Cost per hour for outpatient clinic consultation. 1 h of physician service is calculated per inpatient day.
- The average daily cost for postoperative monitoring and medical surveillance
- The cost of surgery is calculated using the following parameters:
  1. Operating room nurse expenses: Calculated as 2.2 nurses multiplied by the cost per hour and the duration of operating room occupancy.
  2. Anesthesia nurse expenses: Determined as 1.2 nurses multiplied by the cost per hour and the duration of anesthesia administration.
  3. Anesthesiologist fees: Computed as 0.4 physicians multiplied by the cost per hour and the duration of anesthesia administration.
  4. Surgeon fees: Determined by the surgeon’s hourly rate multiplied by the duration of the operation (from incision to closure), plus an additional 30 min allotted for postoperative administrative tasks.
  5. (Cost of other ancillary services such as radiology, laboratory services, specific procedure costs, and implants are embedded in the CPP model).

### 2.6 Ethics

The study was approved by the Regional Ethical Committee of South‐East Norway (REK 429277) and the institution’s data protection officer.

## 3 RESULTS

A total of 47 patients were included in the study; of which 27 were included in the prospective clinical pathway group and 20 in the retrospective control group. The main difference between the intervention group and the control group was the use of NPWT. All patients in the control group were treated with NPWT, in contrast to none in the intervention group. The groups were comparable in terms of age, sex, and comorbidities (Table 1). In the control group, one patient underwent a primary femur amputation, and 12 patients underwent a lower leg amputation, of which five were later converted to a femur amputation. The number of major amputations were reduced from 65% (13/20) to 7.4% (2/27) (*p* < 0.001) after the introduction of the clinical pathway. The number of surgical revisions was reduced from an average of 5.5 to 1.3 per patient, and the length of stay was reduced from 46 to 9 days (Table 2).

**TABLE 1:** Baseline demographics and clinical data.

Variables	Clinical pathway (*n* = 27)	Control group (*n* = 20)
Age, mean (SD) *years*	67 (11)	71 (11)
Males, *n* (%)	25 (93)	16 (80)
BMI, mean (SD*) kg/m* ^*2*^	31.5 (5.0)	28.3 (4.8)
Diabetes type 2, *n* (%)	24 (89)	16 (80)
Hypertension, *n* (%)	9 (33)	2 (10)
IHD, *n* (%)	7 (26)	15 (75)
Kidney failure, *n* (%)	10 (37)	12 (44)
HbA1c, mean (SD) *mmol/mol*	64 (18)	65 (18)
Insulin, *n* (%)	21 (78)	13 (65)
Peripheral artery disease (*n*) (%)	13 (48)	16 (80)
Osteomyelitis *n* (%)	25 (93)	17 (85)
ASA, *n* (%)		
ASA I‐II	5 (19)	3 (15)
ASA III‐IV	22 (81)	17 (85)
Anticoagulants, *n* (%)	14 (70)	17 (85)

Abbreviations: ASA, American Society of Anesthesiologists Classification; BMI, body mass index; HbA1c, hemoglobin A1c; IDH, Ischaemic Heart Disease; SD, standard deviation.

**TABLE 2:** Differences in clinical outcome after 2 years before and after the introduction of a clinical pathway for diabetic foot ulcers.

	Clinical pathway (*n* = 27)	Control group (*n* = 20)	*p*‐value
Length of hospital stay, mean (SD) *days*	9.5 (8.4)	46 (28.0)	<0.001
Number of surgeries per hospital stay, mean (SD)	1.3 (0.8)	5.5 (3.3)	<0.001
Major amputations, *n* (%)	2 (7.4)	13 (65)	<0.001
Mortality within 2 years, *n* (%)	5 (18)	5 (25)	0.591

Abbreviation: SD, standard deviation.

**FIGURE 2:**
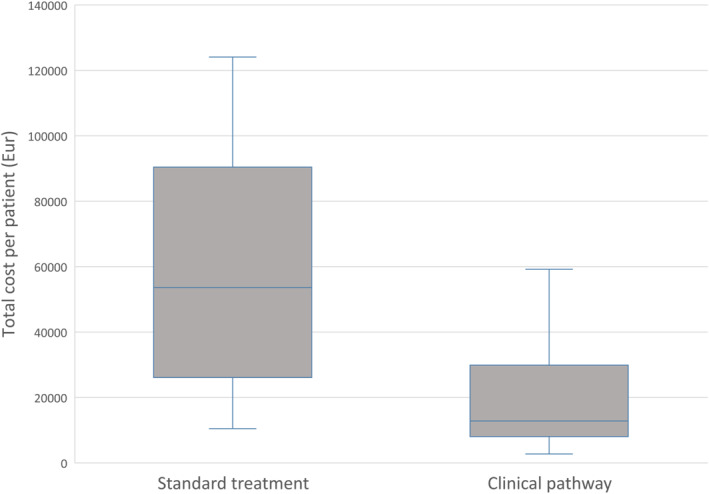
Boxplot demonstrating difference in total cost per patient for two algorithms for diabetes‐related foot ulcer treatment; standard treatment (20 patients) and clinical pathway (27 patients). Cost given in 2022 Euro.

The cost analysis demonstrated that the median total CPP after the introduction of the clinical pathway was reduced by 76% from €538 000 to €129 000 (*p* < 0.001) (Figure 2). The main drivers for cost reduction were reduced hospital stay and fewer operations, while the clinical pathway led to an increased cost in outpatient clinic (Table 3).

**TABLE 3:** Difference in costs before and after the introduction of a clinical pathway for diabetic foot ulcer treatment.

Cost per patient	Clinical pathway	Controls	*p*‐values
Admission	5 722	7 221	0.2
Hospital stay	124 150	401 380	<0.001
Surgery in OR	52 653	184 342	<0.001
Procedures outside OR	2 818	1 477	0.5
Outpatient follow‐up	16 193	7 922	0.04
**Total cost**	**201 536**	**602 342**	**<0.001**

*Note*: All values in 2022 Euros. Bold values are summarized cost (total cost) for each column in 2022 Euros and *p*‐value.

Abbreviation: OR, operation room.

## 4 DISCUSSION

The main finding of this study is that implementation of a clinical pathway for patients suffering from DFU reduced the rate of major lower extremity amputations, length of hospital stays, and number of surgical procedures. Furthermore, the introduction of a systematic and standardized clinical pathway was cost effective, despite increased cost for the outpatient clinic. We found that targeted and fast diagnosis, rapid initiation of treatment, and interdisciplinary collaboration with vascular surgery, infection medicine, and endocrinologist improved the outcome. This is comparable to the results in the studies of M Crane et al. 1999 [15] and Martines Gomez et al. 2014 [16]. Edmonds established a diabetic foot clinic at King’s College Hospital in 1981 [17]. In the first two years of the clinic operating, the number of lower limb amputation amongst people with diabetes at the hospital was reduced by 50%. Patients with diabetic related foot ulcers are among the most complex and vulnerable of all patient populations. Specialized clinics for patients with diabetic foot related ulcers should be multidisciplinary and equipped to coordinate diagnosis, off‐loading, and preventive care; perform revascularization procedures; aggressively treat infections; and manage medical comorbidities.

One of the main changes in the clinical pathway was to re‐introduce standard wound care rather than NPWT following surgical wound revision. We found that NPWT was not mandatory for a good result, and that standard wound care facilitates inspection and monitoring of the DFU at the ward. Our results are in line with the updated guidelines by the International Working Group on Diabetic foot, which recommends that NPWT is not used for non‐surgical DFUs, and that NPWT should only be used as an adjunct therapy to standard of care for the healing of postsurgical diabetes‐related foot wounds [18]. Several RCTs have found that NPWT is superior to standard wound care in terms of higher healing rate at lower cost due to shorter treatment duration and lower number of dressing changes with NPWT [19, 20, 21, 22, 23]. The same studies also report statistically significantly more surgical procedures, dressing changes, and out‐patient visits in the standard wound care group. A large multicenter RCT comparing NPWT with standard moist wound care in Germany found no statistically significant difference in terms of wound healing between the treatment arms [24]. The same authors reported shorter treatment duration and fewer dressing changes with NPWT [23]. However, the traditional wound treatment in that study was not standardized, with dressings applied according to local traditions at the various study sites, which may explain the discrepancy from our results.

Several studies describe a change of dressings twice a day for the standard wound care group, which constitutes a substantial resource use compared to NPWT which requires change of dressings every 48–72 h [19, 22]. In our study dressings were changed once a day for 3–4 days, and then 2–3 times a week, which is comparable to the NPWT group in the RCTs mentioned above.

In our experience, switching from NPWT to standard wound care is more predictable, with less room for treatment failure in a large university clinic where the surgeons on call performing the surgical debridement have a variable degree of experience. There are an abundance of soft tissue and fascial spaces in the feet. Even small amounts of necrotic and infected tissue left inside the sealed NPWT system may cause the infection to spread. There is also a problem with the length of NPWT use, with the wound covered and unavailable for inspection for several days between wound dressing changes. Standard wound treatment allows daily inspection of the wound, which is quite important in the early phase after surgery.

The most important measure is treatment of the underlying illness. Treating the diabetes and its complications will prevent new wounds and potentially reduce mortality. The 2‐year mortality in our study was reduced by a third after the introduction of the clinical pathway (from 30% to 11%), although this did not reach statistical significance.

The 5‐year mortality in patients with DFU are comparable with cancer patients, and there are no good reasons why the treatment for these patients should be less prioritized than cancer treatment [3, 25].

We found a substantial and statistically significant reduction in overall cost per patient after the introduction of the clinical pathway. We noticed that the main cost savings were related to a reduction in hospital stay and surgical procedures. However, the clinical pathway was more expensive for the out‐patient clinic, because the patients are discharged sooner and followed‐up at the out‐patient clinic. Healing of diabetes‐related wounds often takes months, generating numerous out‐patient clinic appointments. Nevertheless, the savings from reduced hospital costs far outweigh the increased need for out‐patient resources. Therefore, a prioritized clinical pathway for treating DFUs is financially profitable, not to mention that it saves limbs. Saving limbs saves lives. Due to the simultaneous introduction of multiple alterations, it is not possible to determine whether standard treatment is superior, inferior, or equivalent to NPWT. To address this, we plan to conduct a randomized controlled trial in which all patients will follow a standardized clinical pathway. Participants will be randomly assigned to receive either standard wound care or NPWT.

### 4.1 Limitations

Our study is limited by a relative low number of patients, which makes conclusions less robust. Many changes were introduced at the same time to create the new clinical pathway, making it difficult to evaluate the individual contribution of each change to the overall results. The increased focus on the patient group may have improved attention and patient care in itself. Furthermore, the study was performed in a single institution, reducing the external validity. The control group is a retrospective cohort, which introduces selection bias as the patients were not randomized. The data from the control group were not systematically collected prospectively, and there are therefore likely missing data and confounders that are unaccounted for when we compare the historical cohort with the prospectively collected data from the clinical pathway. However, our study represents a real world setting in a busy university clinic, which strengthens the clinical relevance.

## 5 CONCLUSION

Implementing a standardized clinical pathway for managing DFUs resulted in significant improvements, including a reduction in major amputations and shorter hospital stays. Discontinuing NPWT after surgical debridement did not adversely affect clinical outcomes. Overall, the new pathway also contributed to lower healthcare costs associated with DFU management.

## AUTHOR CONTRIBUTIONS

**Monica Sailer**: Conceptualization; data curation; formal analysis; funding acquisition; investigation; methodology; writing and reviewing. **Hilde Wergeland**: Analysis and writing. **Per‐Henrik Randsborg**: Conceptualization; formal analysis; funding acquisition; methodology; writing and reviewing.

## CONFLICT OF INTEREST STATEMENT

The authors declare no conflicts of interest relevant to the present study.

## ETHICS STATEMENT

The study was approved by the Regional Ethical Committee of South‐East Norway (REK 429277) and the institution’s data protection officer.

## Data Availability

The data that support the findings of this study are available on request from the corresponding author. The data are not publicly available due to privacy or ethical restrictions.
